# The Endocannabinoid System and Autism Spectrum Disorders: Insights from Animal Models

**DOI:** 10.3390/ijms18091916

**Published:** 2017-09-07

**Authors:** Erica Zamberletti, Marina Gabaglio, Daniela Parolaro

**Affiliations:** 1 Department of Biotechnology and Life Sciences (DBSV), University of Insubria, 21052 Busto Arsizio (VA), Italy; marina.gabaglio@uninsubria.it (M.G.); daniela.parolaro@uninsubria.it (D.P.); 2 Zardi Gori Foundation, 20122 Milan, Italy

**Keywords:** endocannabinoid system, anandamide, cannabinoid type 1 receptor, autism spectrum disorder, animal models

## Abstract

Autism spectrum disorder (ASD) defines a group of neurodevelopmental disorders whose symptoms include impaired communication and social interaction with restricted or repetitive motor movements, frequently associated with general cognitive deficits. Although it is among the most severe chronic childhood disorders in terms of prevalence, morbidity, and impact to the society, no effective treatment for ASD is yet available, possibly because its neurobiological basis is not clearly understood hence specific drugs have not yet been developed. The endocannabinoid (EC) system represents a major neuromodulatory system involved in the regulation of emotional responses, behavioral reactivity to context, and social interaction. Furthermore, the EC system is also affected in conditions often present in subsets of patients diagnosed with ASD, such as seizures, anxiety, intellectual disabilities, and sleep pattern disturbances. Despite the indirect evidence suggestive of an involvement of the EC system in ASD, only a few studies have specifically addressed the role of the EC system in the context of ASD. This review describes the available data on the investigation of the presence of alterations of the EC system as well as the effects of its pharmacological manipulations in animal models of ASD-like behaviors.

## 1. Introduction

Endocannabinoids (ECs) are arachidonic acid-derived compounds that, together with their receptors and the associated metabolic enzymes, constitute the endocannabinoid (EC) system, a neuromodulatory network of lipid signaling pathways [1]. The main ECs are anandamide (AEA) and 2-arachidonoyl glycerol (2-AG) that are generated “on demand” in the post-synaptic cell membrane from phospholipid precursors. The primary biosynthetic enzyme of AEA is *N*-acyl-phosphatidylethanolamine phospholipase D (NAPE-PLD) whereas 2-AG is synthesized by diacylglycerol lipase (DAGL)-α and DAGL-β. Both ECs are rapidly degraded to terminate their actions: AEA is catabolized primarily by fatty acid amide hydrolase (FAAH) while the main 2-AG degrading enzyme is monoacylglycerol lipase (MAGL). ECs exert their effects mainly through the G protein-coupled cannabinoid receptors type 1 (CB1) and type 2 (CB2). Additionally, they are also able to interact with non-CB1/non-CB2 targets, including the transient receptor potential vanilloid type 1 (TRPV1) channel [2], peroxisome proliferator-activated receptor (PPAR)-α, and PPAR-γ [3] as well as the G protein-coupled receptor GPR55 [4].

Alterations of the EC system functionality contribute to the pathogenesis of several psychiatric and neurological disorders [5,6,7]. Indirect evidence for an involvement of the EC system in autism spectrum disorder (ASD) comes from the observation that this system is strongly implicated in the regulation of social and emotional reactivity as well as in the modulation of behaviors that are often altered in ASD, such as learning and memory processes, seizure susceptibility, and circadian rhythm regulation [8,9,10,11,12,13,14,15,16]. Furthermore, human neuroimaging studies revealed associations between polymorphisms in the gene encoding for CB1 receptor, CNR1, and social reward responsivity [17,18,19], suggesting that alterations of CB1 receptors might contribute to deficits in social reward processing associated with ASD. Consistent with this, reduced CB1 receptor expression was found in postmortem brains of individuals with autism [20,21].

Despite these observations being suggestive of an involvement of the EC system in ASD, only a few studies have specifically investigated the role of the EC system in the context of ASD, and our understanding of the EC signaling in ASD is still in its infancy.

The following paragraphs will summarize animal data supporting the fact that (1) the EC system is altered in ASD and (2) pharmacological modulators (Table 1) of the EC system may have a therapeutic potential in ASD. A brief overview of animal models of ASD used to evaluate the role of the EC system as well as a discussion on the possible molecular mechanisms underlying EC-mediated modulation of ASD-like behaviors will be also provided.

## 2. EC-modulation of ASD-Like Behaviors

According to the Fifth Edition of the Diagnostic and Statistical Manual of Mental Disorders (DSM-5), ASD comprises a heterogeneous group of neurological conditions characterized by impaired social communication functions and the presence of restricted, repetitive patterns of behavior or interests that start to emerge in the early developmental period. In subsets of patients, common associated symptoms include seizures, anxiety, intellectual disabilities, motor dysfunctions, altered responsiveness to sensory stimuli, sleep, and metabolic disturbances [22]. Experimental animal models are of fundamental importance for the understanding of the etiology and pathogenesis of ASD as they can provide insights into the pathophysiological processes affected in ASD and allow the development and testing of putative therapeutic agents. Given that ASD is diagnosed by phenotypic behavioral traits, studies in animal models require the evaluation of behaviors relevant to each category of symptoms of human ASD. Indeed, a series of behavioral tests including social interaction and social approach paradigms, olfactory communication, and ultrasonic vocalizations are used to evaluate the impairments of social functioning, whereas behaviors such as compulsive self-grooming and digging are monitored with the intent to investigate the repetitive and stereotyped patterns of behaviors in rodents. Additional tasks allow the evaluation of behaviors relevant to symptoms often associated with ASD, including cognitive deficits, anxiety-like phenotypes, seizures, motor dysfunction, responsiveness to sensory stimuli, and sleep disturbances [23,24].

Several experimental manipulations based on important known causative genetic and environmental factors of ASD have been used to mimic ASD-like phenotypes in animal models. These include the disruption of genes responsible for syndromic forms of ASD, genetic manipulations mimicking human polymorphisms, as well as the exposure to known environmental risk factors. A detailed description of available animal models of ASD is beyond the scope of this review and therefore we refer the reader to recent and comprehensive reviews on this topic [25,26,27]. Hence, we provide a brief overview on both genetic- and environmental-based models that have been exploited to study the involvement and/or the therapeutic potential of the EC system in the context of ASD.

### 2.1. EC System in Genetic-Based Models

#### 2.1.1. Fragile X Mental Retardation (Fmr1) Knockout Mice

Fmr1 mutations lead to a developmental condition called Fragile X Syndrome (FXS). FXS is one of the leading monogenetic causes of ASD and is characterized by intellectual disabilities, developmental delays, anxiety, repetitive behaviors, and physical malformations [28]. Approximately 10%–30% of FXS patients are also diagnosed with autism [29]. The Fmr1 knockout mouse has been extensively characterized as an animal model of FXS. These mice show attention and working memory deficits, hyperactivity, repetitive behaviors, anxiety-related phenotypes, altered social behaviors, and are more susceptible to audiogenic seizures compared to wildtype littermates [30], thus recapitulating many of the features present in FXS patients. Furthermore, Fmr1 knockout mice display structural deficits in dendritic spines [31,32] as well as abnormal glutamatergic [33,34,35] and GABAergic [36,37,38] neurotransmission, indicating that both excitatory and inhibitory defects contribute to circuit dysfunctions in FXS.

The first evidence supporting an involvement of the EC system in FXS came from the studies of Zhang and Alger [39] and Maccarrone et al. [40] which showed that EC-mediated responses at GABAergic synapses of the dorsal striatum and hippocampus are enhanced in Fmr1 knockout mice. Conversely, a study by Jung and colleagues [41] demonstrated the presence of marked deficits in metabotropic glutamate receptor 5 (mGlu5)-dependent 2-AG release and EC-mediated long-term depression (LTD) at excitatory synapses of the forebrain of Fmr1 knockout mice. The authors showed that Fmr1 deletion resulted in an incorrect targeting of DAGLα to dendritic spines, reducing mGlu5-DAGLα functional coupling and ultimately leading to a loss of 2-AG-dependent retrograde signaling at excitatory synapses of the mouse forebrain.

Collectively, these data suggest that alterations of the EC system may contribute to the symptoms of FXS and modulation of the EC signaling may have therapeutic potential in ameliorating some symptoms of FXS. Accordingly, enhancement of 2-AG signaling through administration of JZL184, a pharmacological inhibitor of the 2-AG degrading enzyme MAGL, corrected EC-dependent LTD and improved hyperlocomotion and anxiety-like responses in Fmr1 knockout mice [41].

In addition, modulation of either CB1 or CB2 receptors was shown to ameliorate some of the behavioral signs of Fmr1 knockout mice [42]. In particular, both genetic and pharmacological blockade of CB1 receptors through administration of the CB1 receptor antagonist/inverse agonist rimonabant restored cognitive deficits, seizure susceptibility, and nociceptive desensitization in the mouse model. At the biochemical level, CB1 receptor blockade corrected the overactivation of mTOR signaling and dendritic spine morphology in the hippocampus of Fmr1 knockout mice. Interestingly, treatment with the CB2 receptor antagonist AM630 only affected anxiety-like behavior and audiogenic seizure susceptibility, suggesting a distinct involvement of CB1 and CB2 receptors in the behavioral manifestations of FXS [42]. The beneficial effect of CB1 receptor blockade in the cognitive performance of Fmr1 knockout mice was confirmed in a recent study by Gomis-Gonzales et al. [43]. These authors showed that both low doses of rimonabant and a low dose of the CB1 receptor neutral antagonist NESS0327 prevented cognitive deficits in the Fmr1 knockout mice as measured in the novel object recognition test. Interestingly, the beneficial effect of rimonabant on cognition was associated with the normalization of mGlu-LTD in the hippocampus of Fmr1 knockout mice.

Finally, it has also been shown that modulation of AEA signaling can improve some aspects of the behavioral phenotype in Fmr1 knockout mice. Indeed, acute injection of the FAAH inhibitor URB597 improved aversive memory and anxiety-like behavior in Fmr1 knockout mice, without affecting social behavior [44]. In contrast, Wei and colleagues [45] reported that FAAH blockade via acute URB597 administration completely reversed the social impairment in Fmr1 knockout mice, suggesting that increasing AEA activity at CB1 receptors may exert a prosocial action in animal models of autism. The different pre-treatment times (30 min [44] or 3 h [45]) or the different backgrounds of the animals (C57Bl/6J [44] or FVB/NJ [45]) may account for the discrepant effects of URB597 on social behavior reported in the two studies.

As a whole, available data on Fmr1 knockout mice indicate that imbalanced EC signaling contributes to FXS and correcting EC signaling by targeting different components of the EC system (2-AG, AEA, cannabinoid CB1 and CB2 receptors) might offer an interesting therapeutic strategy for treating some of the clinical manifestations of FXS.

#### 2.1.2. Neuroligin-3 (NLGN3) Mouse Models

NLGNs are postsynaptic cell adhesion molecules required for synaptic function; they orchestrate the maturation and function of both excitatory and inhibitory synapses in the mammalian brain [46,47]. In humans, there are five NLGN isoforms [48]. Mutations of NLGN3 and NLGN4 in particular are associated with X-linked intellectual disability, seizures, and autism behaviors [48]. Arg451Cys (R451C) missense mutation of NLGN3 has been associated with autism in humans [49]. Accordingly, NLGN3 mutant mice carrying the R451C mutation show impaired social interactions and enhanced spatial learning, enhanced synaptic inhibition in the somatosensory cortex and increased excitatory transmission in the hippocampus [50]. In addition, NLGN3 deficiency has been also associated with autism-like behaviors. Indeed, NLGN3 knockout mice show impaired social communication, deficits in social novelty preference, and altered olfactory function, whereas social interaction, prepulse inhibition, and seizure propensity is similar to wildtype littermates [51]. Thus, it appears that NLGN3 mutant mice may only partially model autistic features rather than the global ASD condition. However, these models clearly highlight an association between altered synaptic gene expression and ASD, indicating that synaptic integrity is fundamental for proper brain development and disruption of proteins involved in the regulation of synaptic protein synthesis can lead to ASD [48].

The presence of alterations of the EC system has been investigated both in the NLGN3R451C knockin and the NLGN3 knockout mouse models of ASD-like behaviors. In the hippocampus, both animal models showed a loss of tonic, but not phasic, CB1 receptor-dependent suppression of GABA release at synapses formed by cholecystokinin-expressing (CCK) basket cells to CA1 pyramidal neurons [52]. In addition, Speed and colleagues [53] demonstrated that the increased inhibitory synaptic transmission onto layer II/III cortical pyramidal neurons present in knockin mice expressing the Nlgn3R451C mutation was partly due to a loss of EC signaling through CB1 receptors acting at interneurons other than parvalbumin-positive (PV) or somatostatin-positive (SOM).

Overall, these findings suggest that NLGN3 is required for tonic EC signaling both in the hippocampus and in the somatosensory cortex. Given the common genetic association of the R451C substitution and NLGN3 deletion with ASD, it is possible that disrupted EC signaling could contribute to autism pathophysiology in these mouse models. However, specific pharmacological approaches aimed at modifying EC signaling in the brain of NLGN3 mutant mice are required to establish whether the observed alterations of the EC system directly contribute to the ASD-like phenotype in these mouse models.

#### 2.1.3. BTBR Mouse Model

In addition to the genetically modified rodent models of ASD, several inbred mouse strains incorporate face validity as ASD models, because they display robust and well-replicated social deficits and repetitive behaviors. These inbred strains are considered to be models of idiopathic autism, as their ASD-like phenotype is not caused by known genetic mutations. Among these, of particular interest is the BTBR mouse, which has been the most extensively characterized. BTBR mice display deficits in behavioral tasks modeling the core domains of ASD, including reduced play and social approach behavior [54,55] and restricted-repetitive behaviors [55,56]. These mice also show pronounced cognitive impairments [57] as well as an exaggerated response to stress that is associated with high blood levels of corticosterone [58]. Overall, a number of BTBR behaviors are consistent with autism, and many of the anatomical features in this strain are consistent with those present in autistic patients [59].

Thus far, only one study has investigated the effects of a manipulation of the EC system in the BTBR mouse model [45]. The authors showed that increasing AEA levels via acute administration of URB597 completely reversed the social impairment in the three chamber apparatus, an effect that was not attributable to reduced anxiety. Furthermore, the effect of FAAH inhibition on social approach in BTBR mice was strictly dependent on enhancement of AEA signaling at CB1 receptors, as URB597-mediated recovery of sociability was prevented by concomitant administration of the CB1 receptor antagonist AM251 and it was not associated with alterations in 2-AG contents [45]. This finding in BTBR mice, although preliminary, supports the hypothesis that enhancing AEA signaling could ameliorate ASD-related social impairments.

### 2.2. The EC System in Environmental-Based Models

Although the most commonly suggested etiology of ASD is through the hereditary genetic characteristics identified as high risk genes for ASD, exposure to environmental factors in the prenatal and early postnatal periods imposes a significant contribution to ASD development [60,61]. Relevant environmental manipulations in rodents must be conducted using the same agents that have been correlated with human ASD. Well-known environmental risk factors for ASD in humans are maternal infections and valproic acid (VPA) exposure at the time of neural tube closure.

The EC system has been studied in two environmental-based models: prenatal VPA exposure and postnatal lipopolysaccharide (LPS) administration in rats.

#### 2.2.1. Prenatal VPA Exposure

Among environmental animal models that show both construct and face validity to ASD, the VPA rat model represents an excellent system to test and develop novel behavioral and drug therapies. This model demonstrates many of the structural and behavioral features that can be observed in individuals with autism, thus enabling definition of relevant pathways of developmental dysregulation resulting from environmental manipulation [62]. VPA is an anti-epileptic drug with an identified histone deacetylase inhibitor property [63,64]. Several studies have shown that gestational VPA treatment may cause neural tube defects [65], as well as cognitive impairments [66] in children. This is consistent with animal studies indicating that the offspring of female rats injected with VPA on the 12.5 day of gestation exhibit significantly lower social interaction, increased repetitive/stereotyped behaviors, and early signs of neurodevelopmental impairment with delayed growth and eye opening, olfactory deficit, as well as abnormal responses to painful and non-painful stimuli [67]. Moreover, prenatal exposure to VPA induces sex-related differences in behavior that are greater in males than females [68], thus mimicking the imbalanced gender ratio observed in ASD patients [69]. At the neurochemical level, VPA-exposed rats show hyper-connectivity and hyper-plasticity as well as increased NMDA/AMPA ratio in the medial prefrontal cortex (mPFC) [70,71], and synaptic abnormalities persist into adulthood [72].

The VPA rat model has been extensively used to evaluate the possible implication of the EC system in ASD. Adolescent rats exposed to a single VPA injection at gestational day (GD) 12.5 exhibited reduced mRNA expression of the enzyme primarily responsible for the synthesis of 2-AG DAGLα in the cerebellum and increased activity of the 2-AG-catabolising enzyme MAGL in the hippocampus [73]. mRNA expression of CB1 and CB2 receptors was unaltered [73] but rats prenatally exposed to VPA displayed altered expression of phosphorylated CB1 receptor in the amygdala, hippocampus and dorsal striatum, with no changes in the prefrontal cortex, cerebellum, and nucleus accumbens [74]. In addition, changes in other receptor targets for ECs were observed, namely decreased expression of PPARα and GPR55 in the frontal cortex and PPARγ and GPR55 in the hippocampus [73]. Although no changes in baseline concentrations of AEA and 2-AG were observed, tissue levels of AEA and its congeners oleoylethanolamide (OEA) and palmitoylethanolamide (PEA) were increased in the hippocampus of VPA-exposed rats immediately following social exposure [73], suggesting that prenatal VPA exposure could have altered AEA signaling in response to social stimuli. Accordingly, changes in AEA metabolism from infancy to adulthood have been observed in VPA-exposed rats. Indeed, both reduced expression of NAPE-PLD and increased expression of FAAH were reported in the whole brain of VPA-exposed rats [74].

Of note, enhancing AEA signaling through inhibition of its degradation partially mitigated the behavioral phenotype induced by prenatal VPA exposure. Indeed, systemic administration of the FAAH inhibitor PF3845 at the dose of 10 mg/kg attenuated the deficit in social behavior observed in VPA exposed male animals [75]. In contrast, PF3845 failed to modulate social behavior in female VPA exposed rats [75], suggesting that FAAH inhibition may elicit sexual dimorphic responses in VPA exposed rats. Similarly, URB597 treatment normalized communication abnormalities of VPA-exposed pups in the homing test, and reversed their social deficits in the three-chamber and social play behavior tests [74].

Overall, available data in the VPA rat model suggest that altered AEA-mediated signaling may contribute to communication and social deficits associated with ASD, and support a role of FAAH in the regulation of social behavioral deficits.

#### 2.2.2. Postnatal LPS Injection

Both viral and bacterial infections during pregnancy have been linked to an increased risk to develop ASD in the offspring [76]. Exposure to influenza virus or maternal immune activation (MIA) induced by polyinosine:cytosine, poly(I:C) or LPS in rodents at the time of closure of the neural tube seems to be related to the onset of ASD-like behaviors in the offspring [77]. Hence, the offspring of dams injected with poly I:C between GD 9.5 to 14.5 show impairments in social interaction [78,79], together with deficits in social communication and stereotyped pattern of behavior [80]. In addition, prenatal LPS exposure increases anxiety, decreases social interactions [81] and impairs learning and memory [82] in mice. In rats, gestational LPS exposure also decreases prepulse inhibition in the male offspring [83]. These behavioral phenotypes in the offspring were correlated with synaptic abnormalities, including increased cell density and excitability of pyramidal neurons, enhanced postsynaptic glutamatergic responses to NMDA-induced synaptic plasticity, as well as altered glia reactivity [84]. Taken together, both rat and mouse studies support the association between LPS-induced MIA and ASD phenotypes in offspring.

Although post-natal LPS administration is not recognized as a model of ASD, data collected further suggest that FAAH inhibition may represent a potential approach for the treatment of disorders involving impaired sociability. Alterations of the EC system have been reported in rats after postnatal LPS exposure [85]. Early-life inflammation induced by a single LPS injection at postnatal day (PND) 14 decreased adolescent social play and non-play behavior both in male and in female rats. LPS-induced social deficits were associated with reduced CB1 receptor binding, elevated AEA levels and, surprisingly, increased FAAH activity in the amygdala. Oral administration of the FAAH inhibitor PF-04457845 at the dose of 1 mg/kg prior to the social interaction test normalized LPS-induced alterations in social behavior. A similar improvement was observed after direct PF-04457845 injection into the basolateral amygdala, suggesting that altered AEA signaling in this brain region plays a central role in mediating LPS-induced social impairments at least in females.

## 3. Possible Mechanisms

Many morphological and neurochemical abnormalities have been reported in ASD patients as well as in animal models, reflecting the heterogeneous and complex nature of this group of disorders. Such diversity poses a great challenge and hampers the identification of possible common pathophysiological mechanisms in ASD.

The EC system is involved in the modulation of many of the cellular functions and molecular pathways altered in ASD, such as imbalanced GABAergic and glutamatergic transmission, oxidative stress, altered energy metabolism, immune dysregulation [86,87,88,89,90], hence EC modulation of ASD-like behaviors might arise from its ability to interact with such functions/pathways. However, very little information has been thus far available regarding the possible mechanisms through which the EC system could affect ASD-like behaviors.

For instance, the reported prosocial effects of AEA in animal models of ASD might arise from an interaction with oxytocin, a neuropeptide that promotes parental and social bonding. Indeed, recent evidence has highlighted that oxytocin stimulates AEA release in the nucleus accumbens, a key region for the reinforcing properties of both natural rewards and drugs of abuse, and, importantly, AEA-mediated signaling is required for the prosocial effects of this neuropeptide [91]. This suggests that oxytocin-driven AEA signaling may be defective in ASD; hence, a correction of such deficits supposedly offers a novel strategy to treat the social impairments associated with ASD.

Additionally, it has been shown that the amelioration of cognitive deficits after chronic rimonabant administration in Fmr1 knockout mice was associated with the normalization of the hippocampal mTOR signaling pathway [42], suggesting that the procognitive effects elicited by CB1 receptor blockade may be partly dependent upon restoration of this signaling pathway in the hippocampus. This is consistent with the observation that mTOR signaling is crucially involved in memory consolidation [92], and its genetic modulation prevents some of the pathological features in Fmr1 knockout mice [93]. Importantly, dysregulation of mTOR signaling appears to be a feature common to a subset of ASD [94], suggesting that interaction of the EC system with this molecular pathway might also be involved in ASD conditions other that FXS.

There is also evidence that ECs might modulate ASD symptoms via interaction with immune system cells. Indeed, changes in AEA metabolism and CB2 receptors were observed in peripheral blood mononuclear cells [95] and blood monocyte-derived macrophage cells [96] from autistic patients, suggesting that the EC system could play a role in the immunological dysfunctions associated with ASD.

Of note, converging evidence indicates that ASD is characterized by an excitation/inhibition (E/I) imbalance in selective neuronal circuits that appears to be mainly related to defects of GABAergic signaling in different brain structures [97,98]. As retrograde EC signaling at CB1 receptors is a key regulator of synaptic plasticity both at inhibitory and excitatory synapses in the adult brain [99], dysfunctions of the EC system could sustain ASD phenotypes as a consequence of unbalanced excitatory and inhibitory neurotransmission. On the other hand, considering the regulatory role of the CB1 receptor in the maturation of excitatory and inhibitory neurons [100,101], it is also conceivable that abnormal CB1 receptor signaling at early stages of brain development could have profound consequences on adult brain function, including disruption of the optimal E/I balance and the susceptibility to ASD. Understanding the time course of the alterations of the EC system in animal models of ASD could help to dissect the contribution of this system in the pathogenesis of these neurodevelopmental conditions.

## 4. Conclusions

Although the preclinical findings seem to suggest that pharmacological interventions aimed at modulating the EC system could be beneficial for relieving symptoms associated with ASD (Table 2), their preliminary nature does not allow any definite conclusion to be drawn concerning potential therapeutic exploitations.

Converging data indicate that enhancing AEA signaling through inhibition of its degradation exerts prosocial effects in different animal models of ASD. In addition, CB1 receptor blockade, either acute or chronic, seems to have beneficial effects towards cognitive deficits, at least in mouse models of FXS. Remarkably, in most of the studies, the drugs were administered systemically. However, the alterations of the EC system reported in animal models of ASD (Table 3) appear to be different depending on the brain region considered, possibly suggesting a different contribution to ASD-like symptoms. If so, any potential therapeutic approach is unlikely to involve a single targeted molecule. A better understanding of the possible role of the EC system in the onset and/or progression of ASD symptoms would allow for the evaluation of specific pharmacological interventions that may eventually aid the development of successful drug therapies.

## Figures and Tables

**Table 1:** Pharmacological modulators of the endocannabinoid (EC) system tested in animal models of autism spectrum disorder (ASD).

Drug	Mechanism of Action
URB597 PF-3845 PF-04457845	fatty acid amide hydrolase (FAAH) inhibitors
JZL184	monoacylglycerol lipase (MAGL) inhibitor
Rimonabant	cannabinoid type 1 (CB1) receptor antagonist/inverse agonist
NESS0327	neutral CB1 receptor antagonist
AM630	cannabinoid type 2 (CB2) receptor antagonist/inverse agonist

**Table 2:** Effects of pharmacological manipulations of the endocannabinoid (EC) system in animal models of autism spectrum disorder (ASD).

	Drug	Dose	Model	Outcome	Reference
Anandamide (AEA)	URB597	Acute 0.3 mg/kg	Fragile X Mental Retardation (Fmr1) knockout mice C57BL/6J background	improvement of aversive memory and anxiety-like behaviors	[44]
	URB597	Acute 0.3 mg/kg	Fmr1 knockout mice FVB background	amelioration of social impairments	[45]
	URB597	Acute 0.3 and 1 mg/kg	BTBR mice	amelioration of social impairments	[45]
	URB597	Acute	valproic acid (VPA) exposure in Wistar rats	normalization of communication abnormalities and reversal of social deficits	[74]
	PF-3845	Acute 10 mg/kg	VPA exposure in Sprague-Dawley rats	reversal of social deficits	[75]
	PF-04457845	Acute 1 mg/kg	lipopolysaccharide (LPS) administration in Sprague-Dawley rats	reversal of social deficits	[85]
2-arachidonoyl glycerol (2-AG)	JZL184	Acute 16 mg/kg	Fmr1 knockout mice C57BL/6J background	normalization of locomotion and anxiety-like responses	[41]
Cannabinoid type 1 (CB1) receptor	Rimonabant	Acute 1 mg/kg	Fmr1 knockout mice FVB background	amelioration of cognitive deficits, seizure susceptibility and nociceptive desensitization	[42]
	Rimonabant	Chronic 1 mg/kg	Fmr1 knockout mice FVB background	amelioration of cognitive deficits	[42]
	Rimonabant	Acute 0.3–1 mg/kg	Fmr1 knockout mice FVB background	amelioration of cognitive deficits	[43]
	Rimonabant	Chronic 0.03–1 mg/kg	Fmr1 knockout mice FVB background	amelioration of cognitive deficits	[43]
	NESS0327	Chronic 0.1 mg/kg	Fmr1 knockout mice FVB background	amelioration of cognitive deficits	[43]
Cannabinoid type 2 (CB2) receptor	AM630	Acute 1 mg/kg	Fmr1 knockout mice FVB background	normalization of anxiety-like behavior and audiogenic seizure susceptibility	[42]

**Table 3:** Alterations of the endocannabinoid (EC) system in animal models of autism spectrum disorder (ASD).

Model	Alteration	Brain Area	Reference
Fragile X Mental Retardation (Fmr1) knockout mice	↓ EC-mediated long-term depression (LTD) at excitatory synapses	forebrain	[41]
	↓ EC-mediated LTD at inhibitory synapses	dorsal striatum, hippocampus	[39,40]
Neuroligin (NLGN)3R451C knockin and NLGN3 knockout mice	↓ tonic EC signaling at cholecystokinin-expressing (CCK) basket cell synapses	hippocampus	[52]
Valproic acid (VPA) exposure in rats	↓ tonic EC signaling at interneurons other than parvalbumin (PV)- or somatostatin (SOM)-positive	somatosensory cortex	[53]
	↓ diacylglycerol lipase (DAGL)-α mRNA	cerebellum	[73]
	↑ monoacylglycerol lipase (MAGL) activity and ↓ peroxisome proliferator-activated receptor (PPAR)-γ and G-protein coupled receptor (GPR)55	hippocampus	[73]
	↓ PPARα and GPR55	frontal cortex	[73]
	altered phosphorylation of cannabinoid type 1 (CB1) receptor	amygdala, hippocampus, dorsal striatum	[74]
	↓ *N*-acyl-phosphatidylethanolamine phospholipase D (NAPE PLD) and ↑ fatty acid amide hydrolase (FAAH)	whole brain	[74]
lipopolysaccharide (LPS) administration in rats	↓ CB1 receptor, ↑ anandamide (AEA) levels and ↑ FAAH activity	amygdala	[85]

*↓ = decreased; ↑ = increased.
